# ECONOMIC VALUE OF UNPAID FAMILY CAREGIVER TIME FOLLOWING HOSPITAL DISCHARGE AND AT END-OF-LIFE

**DOI:** 10.1093/geroni/igae098.0465

**Published:** 2024-12-31

**Authors:** Brystana Kaufman, Zhang Wenhan, Sahar Shibeika, Ro Huang, Catherine Vanderboom, Diane Holland, Courtney Van Houtven, Joan Griffin

**Affiliations:** Duke University School of Medicine, Durham, North Carolina, United States; Duke University School of Medicine, Durham, North Carolina, United States; Duke University School of Medicine, Durham, North Carolina, United States; Duke University, Durham, North Carolina, United States; Mayo Clinic, Rochester, Minnesota, United States; Mayo Clinic, Rochester, Minnesota, United States; Duke University School of Medicine, Durham, North Carolina, United States; Mayo Clinic, Rochester, Minnesota, United States

## Abstract

Estimates of patient care costs often overlook the economic value of unpaid caregiving. This study aims to quantify the economic value of unpaid caregiving by rural FCGs. Using data from a multi-site randomized controlled trial evaluating Transitional Palliative Care (TPC) intervention for FCGs, this study quantifies the economic value of family caregiving in the six months following seriously ill care recipients’ hospital discharge using three valuation methods. Caregiving hours were self-reported by FCGs. The proxy cost approach assigned value to FCG hours using the national median home health aide wage. The unadjusted opportunity cost approach assigned value using the national median wage, by sex and education level. The adjusted opportunity cost approach assigned value by work force participation, using opportunity cost if employed and proxy cost otherwise. Of 282 FCGs, 94% were non-Hispanic White, 71% had a college degree, 56% were spouses, and 51% were in the workforce. FCG cost did not differ by treatment arm. FCGs of decedents compared to survivors reported significantly more hours per person-month (392 vs. 272, p< 0.001), resulting in higher median opportunity cost ($12,653 vs. $8,843, p< 0.001), proxy cost ($5,689 vs. $3,955, p< 0.001), and adjusted opportunity cost ($9,490 vs. $6,443, p< 0.001) per person-month. Proxy cost may undervalue FCG time, while opportunity cost may perpetuate labor market inequities. Policymakers must weigh trade-offs of different valuation methods. The high intensity of unpaid post-acute care, especially end-of-life, should be considered when designing policies and interventions to support unpaid FCG due to the economic impact on FCGs and employers.

